# Impact of Canopy Openness on Forest Regeneration and Forest Soil Nutrients

**DOI:** 10.1002/ece3.72735

**Published:** 2025-12-16

**Authors:** Pratik Ojha, Radha Kandel

**Affiliations:** ^1^ Institute of Forestry Tribhuvan University Hetauda Nepal

## Abstract

Canopy structure plays a critical role in regulating forest regeneration and soil nutrient dynamics, yet its specific effects remain insufficiently understood in the subtropical forests of Nepal. This study investigates the impact of canopy openness on forest regeneration and soil nutrients in six *Shorea robusta* ‐dominated mixed community forests within the Chure region of Makawanpur District, Nepal. A total of 90 sample plots for regeneration assessment and 45 soil samples were systematically distributed across three canopy classes—dense (70%–100%), moderate (40%–70%), and open (10%–40%) to evaluate seedling and sapling density, species diversity, and key soil nutrients (N, P, K, SOM, and SOC). The results revealed a distinct trade‐off between regeneration density and species diversity. Seedling density was significantly higher in open canopies, following a clear gradient (open > moderate > dense, *p* < 0.001), whereas sapling density showed no significant difference among canopy classes. In contrast, biodiversity indices (Shannon–Wiener, Simpson's, and equitability) were consistently highest in dense canopies for both seedlings and saplings. Canopy openness also had a strong influence on soil fertility: soil organic matter (SOM), soil organic carbon (SOC), and total nitrogen (N) were all significantly higher in open canopies, supported by positive Spearman correlations (e.g., SOM, *ρ* = 0.51). Phosphorus (P) and potassium (K) levels were unaffected. These findings highlight a trade‐off between regeneration density and diversity, emphasizing the importance of balanced canopy management to sustain both species diversity and soil fertility. The study provides valuable ecological insights for optimizing canopy interventions and promoting sustainable forest management in Nepal's community forests.

## Graphical abstract

This study examines how canopy openness influences tree regeneration and soil nutrient levels in a community‐managed forest in Nepal. The findings highlight the importance of light conditions in silvicultural planning for sustainable forest management.

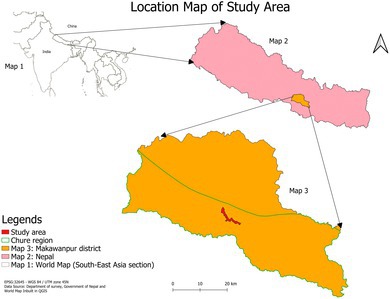

## 1 Introduction

The upper layers of a forest's vegetation, primarily constituted by tree crowns, form the canopy, an essential component of forest ecosystems that significantly impacts biodiversity and ecological processes (Lowman et al. 2019). Canopy structure evolves over time, reflecting complex interactions between plants and their environment (Nakamura et al. 2017). Variations in canopy cover influence light availability, litterfall, nutrient recycling, and microclimatic conditions, all of which are crucial for soil nutrient dynamics and forest regeneration (Tonteri et al. 2016; Liu et al. 2014). The canopy's effect on soil nutrients and regeneration is particularly pronounced in Sal ( *Shorea robusta* Roxb. ex Gaertn.) forests, which are foundational to Nepal's subtropical ecosystems. These forests are characterized by their ecological and economic importance, yet they face significant challenges due to human activities and environmental changes (Pokhrel 2013; Banjade 2012). Soil properties such as pH, nitrogen (N), phosphorus (P), potassium (K), and organic matter play vital roles in determining the regeneration success of *S. robusta* and its associated species (Paudel and Sah 2003; Chaubey and Sharma 2013). Soil organic matter (SOM) and soil organic carbon (SOC) are key indicators of soil health, influencing nutrient availability and carbon sequestration (Lal 2004; Wardle et al. 2004).

Canopy openness—created by natural or anthropogenic interventions—can significantly alter microenvironmental conditions, promoting regeneration by increasing light penetration and modifying soil nutrient dynamics (Doroski et al. 2018). However, excessive canopy opening can lead to adverse effects, such as the proliferation of invasive species, which may inhibit regeneration (Babaasa et al. 2004). Understanding the relationship between canopy cover and regeneration dynamics is critical for sustainable forest management, particularly in community‐managed forests in Nepal, where such interventions are common (Awasthi et al. 2015; Ghimire and Lamichhane 2021).

This study investigated the impact of canopy openness on forest regeneration and soil nutrients in the Chure region of Nepal. It aims to assess the statistical relationship between canopy openness and tree seedling and sapling density while also analyzing tree regeneration diversity and species composition using the Shannon–Weiner and Simpson diversity indices. Additionally, the study evaluates the effects of canopy openness on key soil nutrients, including nitrogen (N), phosphorus (P), potassium (K), soil organic matter (SOM), and soil organic carbon (SOC).

We hypothesize that canopy openness significantly influences the density and diversity of tree regeneration, as well as soil nutrient levels. By addressing these relationships, this study aims to provide insights for forest managers to optimize canopy interventions for enhanced regeneration and ecosystem sustainability.

## 2 Materials and Methods

### 2.1 Study Area

**FIGURE 1:**
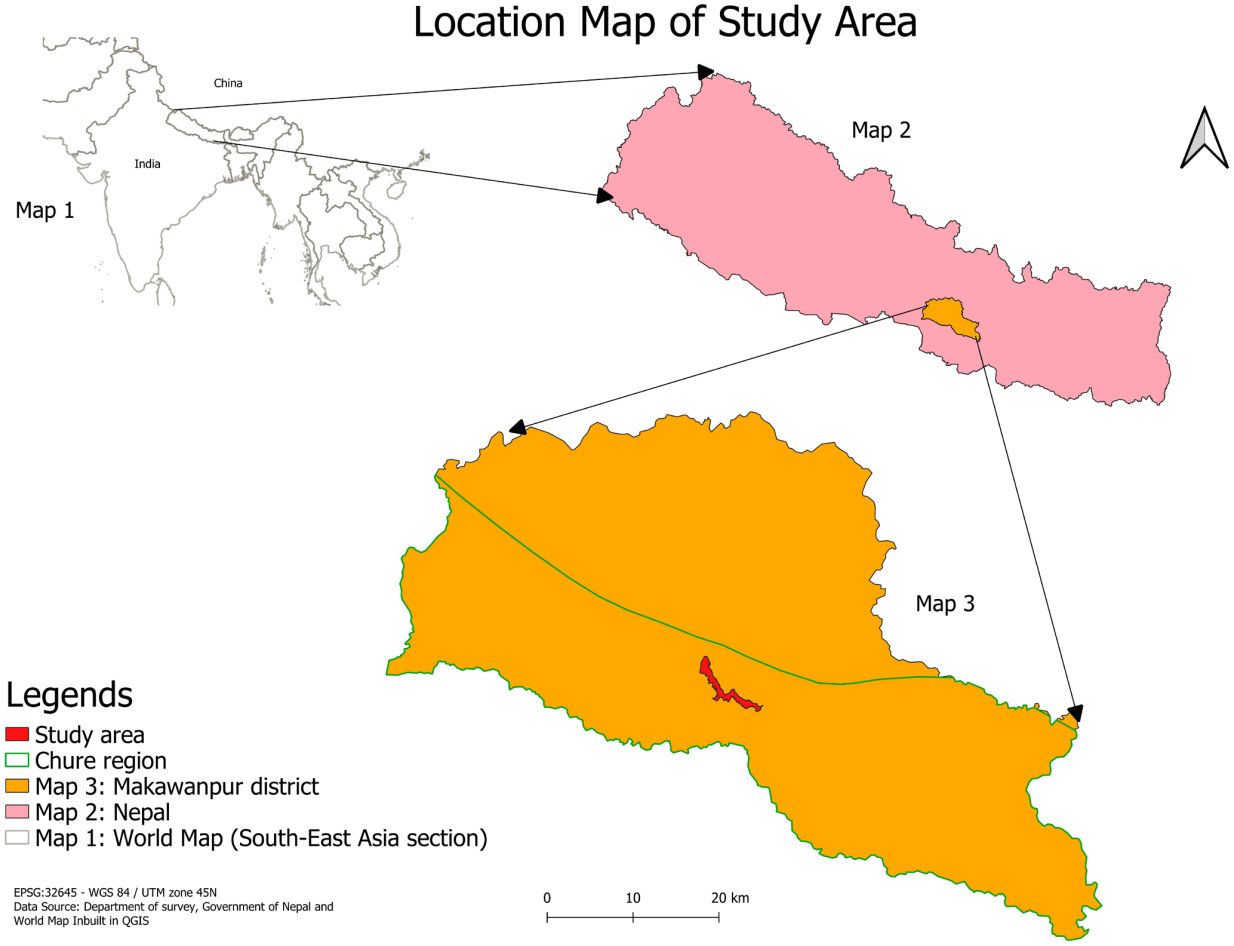
Location map of the study area in the Chure region, Makawanpur district, Nepal. The map illustrates the study area within the Chure region of Makawanpur district, Nepal. It includes three reference maps: (1) a world map highlighting South‐East Asia, (2) a map of Nepal showing the study district, and (3) a detailed map of Makawanpur district with the study area marked in red. *Data source:* Department of Survey, Government of Nepal; map created using QGIS.

The study was conducted in six community forests—Chuchhekhola, Rani Ban, Niureni, Piple Pokhara, Banaskhandi, and Sundar—located in the Chure region of Makawanpur district, Nepal (Figure 1). The area comes under sub‐tropical region with sal ( *S. robusta* ) dominated mixed forest type. The area spans approximately 8 km^2^, with an elevation range of 465–780 m above sea level. The Chure region, Nepal's youngest mountain range, is characterized by its fragile ecosystem, created from riverine sediments during the Himalayan orogeny approximately 40 million years ago (Pokhrel 2013; Bhandari et al. 2016). The region experiences a subtropical climate with distinct wet and dry seasons. The monsoon period from June to September accounts for most of the rainfall. Summers are typically hot, with temperatures exceeding 30°C, while winters are mild, ranging between 20°C and 25°C (Karki et al. 2016). The soils in the study area are primarily Cambisols, which exhibit moderate development with distinct horizon differentiation. These soils, rich in a mix of mineral and organic components, are conducive to plant growth under effective management practices (Gurung 2020).

Three canopy cover classes were delineated within the forests: dense (70%–100% crown cover), moderately dense (40%–70% crown cover), and open (10%–40% crown cover). Selection of the study plots was guided by criteria ensuring minimal anthropogenic interference, including slopes greater than 18°, absence of grazing or silvicultural activities in the past decade as per the community forest inventory guideline 2004 of Nepal, and low fire incidence over the last 20 years, as determined by MODIS fire data and kernel density analysis in ArcGIS (Silverman 2018). The forests are dominated by *S. robusta* , a large, deciduous broadleaf tree, accompanied by species such as Asna (*Terminalia alata Heyne ex Roth*), Jamun ( *Syzygium cumini* (L.) *Skeels*), Seto sirish ( *Albizia procera* (*Roxb*.) *Benth*.), and Pahari (*Trewia nudiflora L*.) (Kc 2007). These forests play a crucial ecological and economic role, supporting biodiversity and providing resources for local communities.

### 2.2 Method of Data Collection

#### 2.2.1 Study Design and Sampling

**FIGURE 2:**
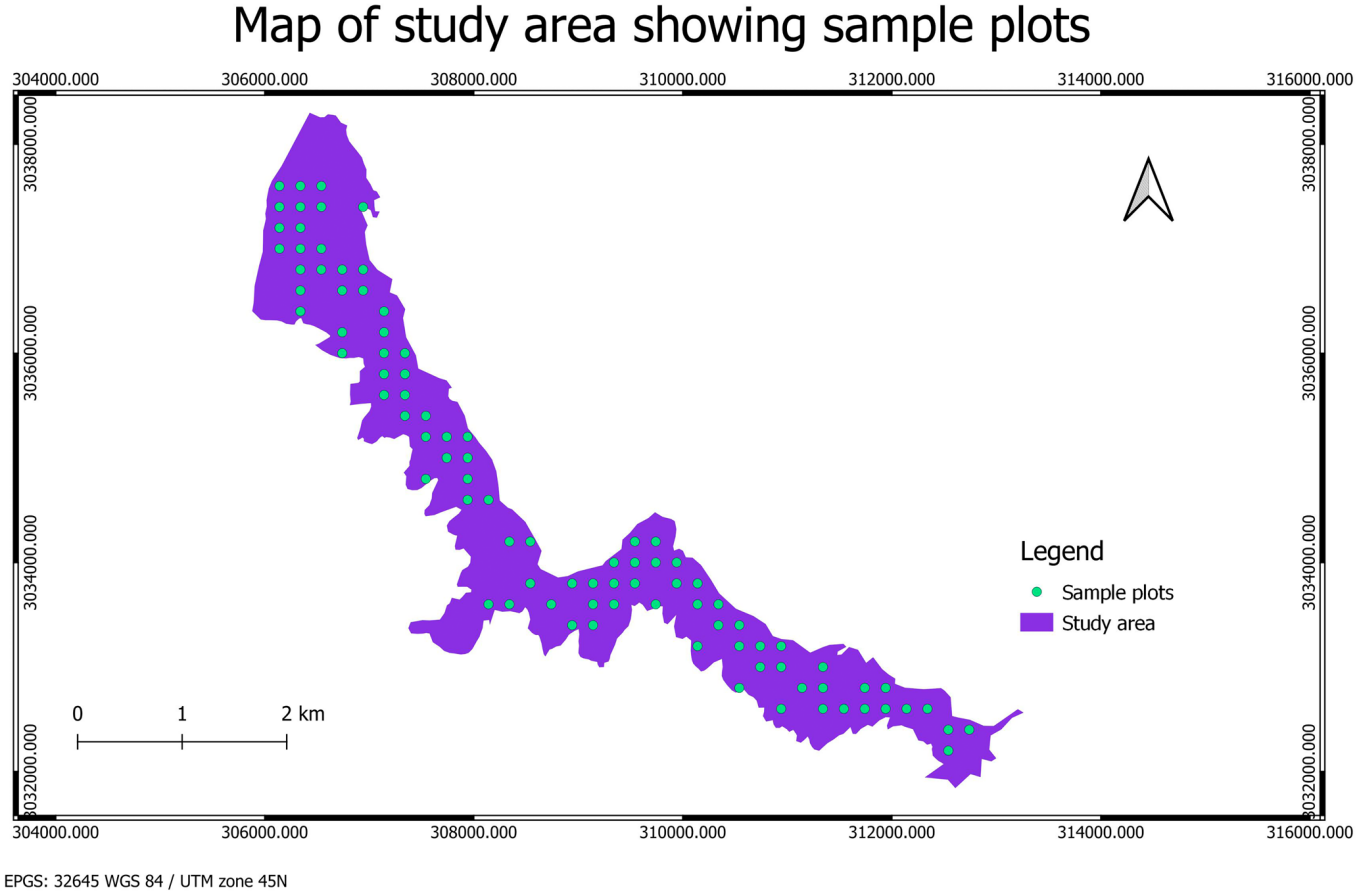
Sample point distribution in study area. This figure illustrates the study area with sample plots marked in green. The study area is highlighted in purple. The coordinate system used is EPSG: 32645 (WGS 84/UTM zone 45N). *Data source:* Field survey and GIS analysis.

Data collection was conducted in six community forests (CFs) located in the Chure region of the Makawanpur district, Nepal. To ensure minimal human interference, regions with slopes greater than 18°, low fire density (identified through 20‐year fire incident data from NASA's FIRMS), and no recent silvicultural operations were selected. The sampling intensity was set at 0.1%, following the Community Forest Inventory Guideline (2004) of Nepal. Systematic sampling was employed using the ArcGIS fishnet tool, resulting in 90 sample plots as seen in Figure 2. A densiometer was used to measure the crown's openness. The protocol for collecting the data was referenced from Lemmon (1957). 90 sample plots were then distributed across dense (27 plots), moderately dense (32 plots), and open (31 plots) canopy strata.

#### 2.2.2 Regeneration Data

To assess vegetation regeneration, concentric circular plots of 10 m^2^ and 25 m^2^ were established to monitor seedlings and saplings, respectively. Seedlings, defined as plants less than 1.3 m in height, were counted within the 10 m^2^ plots, providing insights into early‐stage plant recruitment. Saplings, defined as plants greater than 1.3 m in height with a diameter at breast height (DBH) less than 5 cm, were recorded in the 25 m^2^ plots, indicating the transition to juvenile growth. This method allows for a comprehensive assessment of forest regeneration, helping to gauge the plant community's sustainability and future growth potential (FRA 2022).

#### 2.2.3 Soil Sampling

Soil sampling was conducted across the three canopy strata to assess soil properties in relation to vegetation growth and regeneration. A total of 45 individual soil samples were collected, with 15 samples taken from each canopy stratum (dense, moderately dense, and open). At each of the 45 sampling points, the layer of fresh, undecomposed leaves and twigs on the surface was first manually brushed aside. Then, samples were collected from the surface layer (0–20 cm depth), the primary zone for root activity and nutrient uptake. Each sample was extracted using a cylindrical corer (107.5 cm^3^ volume), and the soil collected from the 0–20 cm profile within the corer was treated as an internally mixed, single sample. Crucially, each of these 45 samples was bagged, labeled, and analyzed individually. These samples were then sent to the Regional Soil Laboratory in Hetauda, where they were analyzed to measure critical soil nutrient levels, including total nitrogen (N), available phosphorus (P), exchangeable potassium (K), soil organic matter (SOM), and soil organic carbon (SOC). The laboratory employed several established analytical methods, including the Walkey–Black method for soil organic matter, the Olsen method for phosphorus, and flame photometry for potassium analysis, following the protocols outlined by Jahn et al. (2006). These analyses provide valuable data on soil fertility and nutrient availability, which are crucial for understanding the ecological dynamics and potential for plant growth in the study area.

#### 2.2.4 Secondary Data

Secondary data for the study was collected from two primary sources: NASA FIRMS and the Aster DEM dataset. NASA's Fire Information for Resource Management System (FIRMS) provided historical forest fire data from 2003 to 2021, sourced from the Moderate Resolution Imaging Spectroradiometer (MODIS) sensor on NASA's Aqua and Terra satellites. This dataset offers fire records and insights into fire occurrences, spatial distribution, and frequency. Additionally, the Visible Infrared Imaging Radiometer Suite (VIIRS) Fire Data from NASA FIRMS (2002) contributed further fire incident data. The Aster DEM dataset, obtained from the Vertex/Alaska Satellite Facility, was used to analyze terrain characteristics such as aspect, elevation, and slope. This raster format dataset, derived from remote sensing technology, aids in understanding terrain variations and gradients. Both datasets were essential for assessing the fire risk in the study area. Complementing these, quantitative data such as the boundary (GPS points) of Community Forests (CFs) were acquired from community forest operational plans. Relevant information was gathered through a review of both published and unpublished documents, including journals, websites, and government records from the Department of Forest (DoF) and Department of Forest Research and Survey (DFRS), as well as visits to nearby forest offices. These sources provided crucial information for evaluating the study area's fire risk and other relevant ecological aspects.

### 2.3 Statistical Analysis

Data were analyzed using Microsoft Excel and RStudio (ver. 2024.04.01). ANOVA was applied to assess variations in regeneration density and soil nutrients across canopy classes. Biodiversity metrics such as Shannon–Weiner Index, Simpson Diversity Index, and Equitability Index were calculated to evaluate species diversity and distribution which are described as follows.

#### 2.3.1 Simpson's Index (*D*)

It was calculated to measure the probability that two individuals randomly selected from a sample will belong to the same species (or some category other than species).

$$D=\sum n\;\left(n-1\right)/N-1$$

where *n* = the total number of organisms of a particular species, *N* = the total number of organisms of all species.

#### 2.3.2 Shannon–Weiner Diversity Index (*H*′)

It was calculated to compare the diversity of regeneration among the varying canopy cover plots.

$$H^{\prime }=\sum -\left(\mathrm{Pi}*\ln\;\mathrm{Pi}\right)$$

where *H*′ = index of species diversity, Pi = relative abundance of each species, that is, the proportion of individuals of a given species relative to the total of individuals in the community.

#### 2.3.3 Shannon's Equitability Index (*E*_*H*_)

It was calculated to measure how evenly individuals are distributed among the different species in a community, providing a value from 0 to 1 where 1 represents a perfectly balanced distribution.

$$E_{H}=H^{\prime }/\ln\left(S\right)$$

where *H*′ is the Shannon Diversity Index, which is calculated first; *S* is the total number of species in the community (species richness), and ln is the natural logarithm.

A Spearman's rank correlation analysis was conducted to determine the strength, direction, and significance of the association between canopy openness (%) and the soil variables (SOM, N, SOC, P, and K). The resulting correlation coefficient (*ρ*) and *p*‐value were used to assess the relationship, with significance accepted at the *p* < 0.05 level.

## 3 Results

### 3.1 Regeneration Assessment

The study revealed significant variation in tree regeneration density across the three canopy strata (Table 1). Seedling density was highest under open canopy conditions (18,871 ± 5208 ha^−1^), followed by moderately dense (14,281 ± 3303 ha^−1^) and dense canopy strata (11,185 ± 2587 ha^−1^). Analysis of variance (ANOVA) indicated that these differences were statistically significant (*F* = 28.65, *p* < 0.001). Tukey's post hoc comparisons further confirmed that seedling densities differed significantly among all canopy classes, with open > moderate > dense. In contrast, sapling densities showed little variation among canopy types, with mean values of 3226 ± 709, 3200 ± 1325, and 2889 ± 1043 ha^−1^ for open, moderately dense, and dense canopies, respectively, and no statistically significant differences (*F* = 0.88, *p* = 0.41). These results indicate that canopy openness exerts a strong positive influence on seedling establishment but has a limited effect on sapling density.

**TABLE 1:** Mean regeneration density (per hectare) and statistical summary across canopy strata.

Regeneration type	Canopy class	Mean ± SD (per ha)	*F*	*p*	Significant pairwise differences (Tukey test)
Seedlings	Open	18,871 ± 5212	28.65	< 0.001	Open > moderate > close
	Moderate	14,281 ± 3305			
	Close	11,185 ± 2587			
Saplings	Open	3226 ± 707	0.88	0.41	ns (no significant differences)
	Moderate	3200 ± 1325			
	Close	2889 ± 1043			

*Note:* The table shows that seedling density differed significantly among canopy classes (*p* < 0.001), while sapling density showed no significant variation (*p* = 0.41).

Abbreviation: ns, not significant.

Chi‐squared analysis further indicated significant differences in regeneration density across canopy classes (*χ* ^2^ = 226.68, *p* < 0.05). Despite this, a low Cramer's *V* value (0.065) suggested a weak practical association between canopy openness and regeneration density.

### 3.2 Tree Regeneration Diversity and Composition

The diversity and evenness of tree regeneration varied across the three canopy strata, as summarized in Table 2. A clear trend was observed for both seedlings and saplings, with the highest values for all diversity indices consistently found in the dense canopy areas. This indicates that dense canopies support a more balanced and diverse species composition in the early stages of regeneration.

**TABLE 2:** Diversity indices for tree regeneration across different canopy strata.

Regeneration type	Canopy strata	Shannon–Weiner Index (*H*′)	Simpson's Index (*D*)	Shannon's equitability (*E*_*H*_)
Seedling	Open	1.86	0.69	0.63
	Moderate	1.78	0.66	0.61
	Dense	2.08	0.76	0.71
Sapling	Open	2.4	0.83	0.82
	Moderate	2.48	0.86	0.84
	Dense	2.51	0.86	0.85

*Note:* The table shows values for the Shannon–Weiner Diversity Index (*H*′), Simpson's Diversity Index (*D*), and Shannon's Equitability Index (*E*_*H*_) for seedlings and saplings.

**FIGURE 3:**
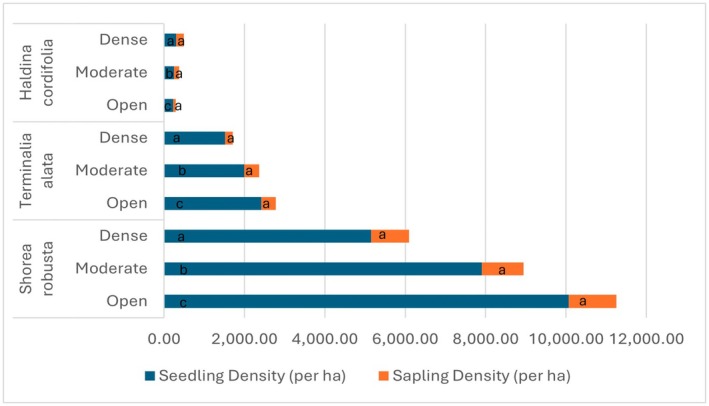
Seedling and sapling density of key tree species under different canopy conditions. *Shorea robusta* and *Terminalia alata* are light‐demanding species, while *Haldina cordifolia* is shade‐tolerant. Data represent seedling and sapling densities (per hectare). For a given species and regeneration type, bars labeled with different letters (a, b, c) are statistically different from each other, while bars sharing the same letter (a) are not significantly different (*p* > 0.05, Tukey's HSD test).

Species Composition and Functional Traits Analysis of individual species revealed distinct patterns in response to canopy openness, as seen in Figure 3. For seedlings, statistically significant differences among canopy classes are indicated by different letters (a, b, c); in contrast, the same letter (a) is used for all sapling groups to show a lack of significant difference. Light‐demanding species such as *S. robusta* and *T. alata* were most abundant in open canopy strata. For example, *S. robusta* seedlings had densities of 10,064 per ha in open canopy strata, compared to 7906 per ha in moderately dense strata and 5148 per ha in dense strata. Similarly, *T. alata* seedlings exhibited higher densities in open strata (2419 per ha) than in dense strata (1518 per ha). Conversely, shade‐tolerant species like Haldu (*Haldina cordifolia* (*Roxb*.) *Ridsdale*) were more prevalent in dense canopy strata. *H. cordifolia* seedlings had higher densities in dense strata (296 per ha) compared to open strata (226 per ha). These trends reflect the specific silvicultural preferences of tree species, with light‐demanding species thriving in open conditions and shade‐tolerant species favoring dense canopy covers. Overall, while open canopy conditions favor the regeneration of dominant light‐demanding species, dense canopies provide a more equitable environment that supports a broader range of species, contributing to overall ecosystem stability.

### 3.3 Soil Nutrient Analysis

Canopy openness had a significant influence on SOM, SOC, and N levels, as evidenced by ANOVA results (*p* < 0.05). Canopy openness significantly boosted the nutritional quality of the soil, particularly in terms of SOM%, SOC%, and N% content reflecting enhanced nutrient cycling under increased light and organic input conditions. In contrast, P and K levels showed no significant differences among canopy classes (*p* > 0.05) as seen in Table 3.

**TABLE 3:** Analysis of variance (ANOVA) results for soil properties under different canopy strata.

Soil property	*F*	*p*	Significance
SOM	13.2	< 0.05	*Significant*
N	13.2	< 0.05	*Significant*
P	1.01	0.37	ns
SOC	12.2	< 0.05	*Significant*
K	2.66	0.08	ns

*Note:* The table presents ANOVA results for various soil properties: Soil Organic Matter (SOM), Nitrogen (N), Phosphorus (P), Soil Organic Carbon (SOC), and Potassium (K). The significance (Sig.) values indicate whether differences among groups are statistically significant (*p* < 0.05). Significant differences were observed for SOM, N, and SOC, while P and K did not show statistically significant variation among groups.

To directly assess the relationship between canopy openness and soil nutrients, a Spearman correlation analysis was performed (Table 4). The results show a significant and positive correlation between canopy openness and the levels of SOM, SOC, and N. No significant correlation was found for phosphorus or potassium.

**TABLE 4:** Spearman's correlation between canopy openness and soil properties.

Soil property	Spearman's correlation coefficient (*ρ*)	*p*
SOM	0.51	< 0.001
N	0.442	0.002
SOC	0.484	0.001
P	−0.149	0.329
K	0.207	0.174

*Note:* The table shows the correlation coefficient (*ρ*) and the *p*‐value for the relationship between the percentage of canopy openness and key soil variables (*n* = 45).

Canopy openness had a significant influence on SOM, SOC, and N levels, as evidenced by ANOVA results (*p* < 0.05). To determine which specific canopy classes differed from one another, a Tukey's HSD post hoc test was performed. The results of these pairwise comparisons are presented in Table 5.

**TABLE 5:** Multiple comparison of soil properties using Tukey's HSD test.

Soil property	Canopy type	Mean ± SD	*F*	*p*	Significant pairwise differences (Tukey test)
SOM	Open	3.36 ± 0.63	13.2	< 0.05	Open > medium > close
	Medium	2.55 ± 0.74			
	Close	2.11 ± 0.84			
Nitrogen	Open	0.16 ± 0.02	13.2	< 0.05	Open > medium > close
	Medium	0.12 ± 0.03			
	Close	0.10 ± 0.03			
SOC	Open	1.96 ± 0.38	12.2	< 0.05	Open > medium > close
	Medium	1.53 ± 0.47			
	Close	1.25 ± 0.46			

*Note:* The table shows the *p*‐values for pairwise comparisons of soil properties that were found to be significant in the ANOVA test. A *p*‐value < 0.05 indicates a significant difference between the groups.

## 4 Discussion

### 4.1 Influence of Canopy Openness on Regeneration and Diversity

Canopy openness plays a pivotal role in tree regeneration and species diversity. The findings of this study revealed that seedling density significantly increased with canopy openness, whereas sapling density did not show a significant relationship with canopy cover (Table 1). This result aligns with previous studies that have demonstrated the positive effect of light availability on seedling establishment and growth, particularly for light‐demanding species such as *S. robusta* (Baral and Ghimire 2020; Gautam and Devoe 2006). Open canopy strata provided optimal conditions for seedling establishment, including reduced competition and higher light penetration. However, species diversity and evenness showed the opposite trend, consistently peaking in dense canopy strata for both seedlings and saplings (Table 2). This suggests that while creating large gaps is an effective strategy to boost the numbers of a target species, it may lead to a less diverse and more homogenous forest understory. Dense canopies, by fostering a more stable microclimate, appear to support a wider and more balanced community of regenerating species. The low Cramer's *V* value for regeneration density suggests that although statistically significant, the relationship between canopy classes and regeneration density is not strong. This may be due to the interplay of other ecological factors, such as soil moisture and nutrient availability, which also influence regeneration dynamics. These findings are consistent with the observations of Ghimire and Lamichhane (2021), who noted that forest management practices enhancing light availability significantly improved regeneration but required a balance to prevent excessive herbaceous growth.

### 4.2 Species‐Specific Responses to Canopy Openness

The species‐specific response to canopy conditions was evident. Light‐demanding species such as *S. robusta* and *T. alata* thrived under open canopy conditions, while shade‐tolerant species such as *H. cordifolia* preferred denser canopy strata. This observation underscores the need for species‐specific forest management approaches that consider the ecological preferences of target species to optimize regeneration outcomes. The diversity indices further supported this pattern. The higher diversity indices observed under dense canopies (Table 2) indicate a more balanced species composition and reduced dominance by any single species. In contrast, open canopy strata, though rich in individuals, were strongly dominated by *S. robusta* . Dense canopies exhibited higher Shannon–Wiener and Simpson diversity values for saplings, reflecting greater evenness and community stability, whereas open canopies showed higher species richness but lower evenness, emphasizing the dominance of pioneer species such as *S. robusta* . These findings corroborate earlier studies, suggesting that dense canopies foster greater diversity by providing a range of niches, while open canopies promote the establishment of early‐successional species (Zellweger et al. 2020).

### 4.3 Impact of Canopy Openness on Soil Nutrients

Our study demonstrated a strong, positive, and seemingly linear relationship between canopy openness and key soil nutrients. The initial ANOVA results (Table 3) were strongly corroborated by a Spearman's correlation analysis (Table 4) and a Tukey's post hoc test (Table 5), which consistently showed the highest concentrations of SOM, SOC, and N in open canopies. A straightforward interpretation is that increased solar radiation elevates soil temperatures, which stimulates the microbial decomposition of leaf litter and accelerates the mineralization of organic matter (Mladenoff 1987; Schliemann and Bockheim 2011). However, this linear trend requires a more critical discussion in the era of climate change. The reviewer correctly notes that the processes of mineralization and humification are fundamentally dependent on a favorable balance of temperature and soil moisture to maintain high enzymatic activity. While moderate canopy gaps can create optimal warming, excessive canopy opening can lead to extreme solar radiation (insolation) on the forest floor. This can drastically increase soil temperatures and lead to rapid soil drying, creating conditions that inhibit or even kill the microbial communities responsible for decomposition, consequently slowing down mineralization (Sariyildiz and Anderson 2003; Gray et al. 2002).

A more complete ecological model would predict a “hump‐shaped” relationship between canopy openness and soil fertility. While our findings show a positive linear trend, this is likely because the “open” sites in our study (10%–40% canopy cover) represent a beneficial level of disturbance that has not yet crossed a negative, moisture‐limited threshold. In reality, the fertility increase is not infinite; while moderate gaps enhance nutrient cycling, very large clearings can lead to excessive soil drying and a deterioration of soil health (Bauhus et al. 2004). Furthermore, the significant increase in Nitrogen without a corresponding rise in Phosphorus and Potassium could create nutrient imbalances. Under the increasing threat of drought due to climate change, this excess nitrogen could become a limiting factor for stand stability rather than a benefit (Cao et al. 2023). This highlights that while creating gaps can enhance nutrient cycling, their size must be carefully managed to avoid the detrimental effects of excessive soil drying and potential nutrient leaching (Ghimire and Lamichhane 2021).

### 4.4 Limitations and Recommendations

This study provides an important snapshot of how canopy openness influences forest regeneration and soil nutrients under relatively undisturbed conditions. However, a few limitations should be noted. Because our data were collected at a single point in time, the long‐term success of the seedlings observed in open areas remains uncertain. Furthermore, our study did not measure key underlying variables that drive these ecological processes. Factors such as microclimate (e.g., soil moisture, temperature), underlying geology, and direct measures of microbial or enzymatic activity would provide a more complete mechanistic understanding of our results.

By focusing on forests with minimal human activity, this research establishes a valuable ecological baseline. Future studies should extend this work to community‐managed forests where practices such as selective harvesting and grazing occur, which would clarify how human activities modify these relationships. Additionally, future research that incorporates the measurement of these unmeasured soil and microclimate variables would be a crucial next step in explaining the patterns observed.

## 5 Conclusion

Our study demonstrates that canopy openness is a key ecological factor regulating regeneration dynamics and soil nutrient composition in *S. robusta‐dominated* mixed forests of Nepal. A distinct trade‐off was observed between regeneration density and species diversity: open canopies promoted higher seedling densities, particularly of dominant light‐demanding species, while dense canopies supported greater species diversity and evenness. Canopy openness also enhanced soil fertility by increasing the concentrations of soil organic matter, organic carbon, and nitrogen. These results suggest that while canopy openings can be strategically used to encourage regeneration of target species, maintaining a heterogeneous canopy structure is essential for sustaining overall forest biodiversity, soil health, and ecosystem resilience.

## Author Contributions

**Pratik Ojha:** conceptualization (lead), data curation (lead), formal analysis (lead), investigation (lead), methodology (lead), project administration (equal), resources (lead), software (lead), validation (lead), visualization (lead), writing – original draft (lead), writing – review and editing (lead). **Radha Kandel:** formal analysis (supporting), resources (equal), software (supporting), writing – review and editing (equal).

## Funding

The authors have nothing to report.

## Ethics Statement

We affirm that this manuscript adheres to the highest standards of ethical behavior in research and publication. We have followed the ethical guidelines outlined by the journal, ensuring that all parties involved in the submission, review, and publication process have acted with integrity and respect.

## Conflicts of Interest

The authors declare no conflicts of interest.

## Data Availability

The datasets analyzed during this study are available as: Ojha, P. (2025). Main Dataset for Canopy Openness and Forest Regeneration Study (MSExcel) [Data set]. *Zenodo*. https://doi.org/10.5281/zenodo.15631126. Ojha, P. (2025). Impact of Canopy Openness in Soil Nutrients [Data set]. *Zenodo*. https://doi.org/10.5281/zenodo.17479290.
